# Matrix Stiffness-Upregulated MicroRNA-17-5p Attenuates the Intervention Effects of Metformin on HCC Invasion and Metastasis by Targeting the PTEN/PI3K/Akt Pathway

**DOI:** 10.3389/fonc.2020.01563

**Published:** 2020-08-19

**Authors:** Xiangyu Gao, Xiaona Qiao, Xiaoxia Xing, Jinya Huang, Jiali Qian, Yi Wang, Yawen Zhang, Xi Zhang, Miao Li, Jiefeng Cui, Yehong Yang

**Affiliations:** ^1^Department of Endocrinology, Huashan Hospital, Fudan University, Shanghai, China; ^2^Liver Cancer Institute, Zhongshan Hospital, Fudan University & Key Laboratory of Carcinogenesis and Cancer Invasion, Ministry of Education, Shanghai, China

**Keywords:** hepatocellular carcinoma, metformin, matrix stiffness, resistance, biomechanics

## Abstract

**Background:**

Metformin, a traditional first-line anti-hyperglycemic agent for diabetes, recently exhibits better antitumor effect in hepatocellular carcinoma (HCC). However, its resistance and tolerance mechanism in HCC remains largely unknown. Here, we investigated whether increased matrix stiffness attenuated the intervention effects of metformin on HCC invasion and metastasis, and explored its underlying molecular mechanism.

**Methods:**

FN-coated gel substrates with 6, 10, and 16 kPa, which simulated the stiffness of normal, fibrotic, and cirrhotic liver tissues respectively, were established to evaluate matrix stiffness-mediated effects on HCC cells. Alterations in morphology, proliferation, motility, and invasive/metastatic-associated genes (PTEN, MMP2, MMP9) of HCC cells grown on different-stiffness substrates were comparatively analyzed before and after metformin intervention. Subsequently, the underlying molecular mechanism by which higher matrix stiffness attenuates antitumor effects of metformin in HCC was further elucidated.

**Results:**

Metformin significantly inhibited proliferation, migration, and invasion of HCC cells. Compared with the controls on lower-stiffness substrate, HCC cells grown on higher-stiffness substrate exhibited an obvious resistance to intervention effects of metformin on proliferation, migration, invasion and metastasis. High stiffness stimulation significantly activated the miR-17-5p/PTEN/PI3K/Akt signaling pathway in HCC cells via integrin β1 and in turn resulted in MMP2 and MMP9 upregulation. Meanwhile, integrin β1 knockdown or PI3K inhibitor partially reversed the activation of the above signaling molecules. For HCC cells grown on the same-stiffness substrate, metformin remarkably upregulated PTEN expression and suppressed the activation of the PI3K/Akt/MMP pathway, but no effect on integrin β1 expression. Importantly, the increase in fold of PTEN expression and decrease in folds of Akt phosphorylation level and MMP2 and MMP9 expressions in the treated HCC cells with metformin on 16-kPa stiffness substrate were evidently weakened compared with those in the controls on the 6-kPa stiffness substrate.

**Conclusions:**

Increased matrix stiffness significantly attenuates the inhibitory effect of metformin on HCC invasion and metastasis, and a common pathway of PTEN/PI3K/Akt/MMPs activated by mechanical stiffness signal and inactivated by metformin contributes to matrix stiffness-caused metformin resistance. To the best of our knowledge, this is the first report to clarify the mechanism of metformin intervention resistance from the perspective of tumor biophysical microenvironment.

## Introduction

“New uses of old drugs” has gradually become a new highlight in therapy research of hepatocellular carcinoma (HCC). Metformin, as a traditional first-line anti-hypoglycemic agent for diabetes, recently exhibits better antitumor effects in HCC. Type 2 diabetes patients treated with metformin have a lower risk for HCC development compared with those taking other antidiabetic drugs in a large population-based prospective clinical epidemiological survey (1). Besides, metformin alone or combined with other drugs obviously counteracts malignant features of HCC through multiple mechanisms (2–5), suggesting an obvious antitumor role of metformin in HCC development and progression. Similar to other chemotherapy drugs, metformin may be also developed into drug tolerance and resistance in HCC during long-term treatment. However, to date little is known about the underlying molecular mechanisms of metformin resistance and tolerance in HCC. Generally, drug resistance and tolerance frequently attribute to gene mutation of tumor cells and their surrounding microenvironment alteration. A recent study demonstrates that colorectal cancer (CRC) cells could increase DNA mutation to evade targeted therapies (6). Other studies also reveal that gene KRAS mutation-mediated acquired resistance occurs in CRC (7, 8). NOXA mRNA destabilization/MCL-1 adaptation limits apoptotic response of multiple cancer cell lineages in the course of targeted therapy (9). Additionally, metabolic reprogramming also participates in the regulation of tumor resistance to conventional chemotherapy (10, 11). Most of these studies on explaining therapeutic resistance mainly focus on biochemical and metabolic factors but pay little attention to biophysical and biomechanical cues within the tumor microenvironment. Higher matrix stiffness, as a mechanical feature of solid tumor, can remarkably strengthen the malignant characteristics of HCC cells such as stemness (12), proliferation (13), invasion and metastasis (14), chemoresistance (15), pre-metastatic niche formation (16), and epithelial–mesenchymal transition (EMT) (17). Additionally, HCC cells under higher stiffness stimulation present chemotherapeutic resistance to cisplatin (15). Analysis of a 3D HCC model with controllable matrix stiffness also reveals that higher matrix stiffness results in the strongest chemotherapeutic resistance to paclitaxel, 5-FU, and cisplatin in HCC (18). Except that, our previous work also implies that higher matrix stiffness weakens oxaliplatin-induced apoptosis in HCC cells (12). Accordingly, we speculate that high matrix stiffness might attenuate the intervention effect of metformin on HCC invasion and metastasis. Unlike other studies about biochemical cue-caused chemotherapeutic resistance, this study aimed to explore the potential roles of biophysical mechanical signals in metformin intervention resistance on HCC invasion and metastasis and its underlying molecular mechanism.

## Materials and Methods

### Preparation of FN-Coated Polyacrylamide Gel Substrates With Stiffness 6, 10, and 16 kPa *in vitro*

FN-coated polyacrylamide gel substrates with stiffness 6, 10, and 16 kPa were respectively established as the method described previously except for the coated matrix protein (13). A gel substrate with a diameter of 6 cm was coated with 320 μl fibronectin solution (0.17 mg/ml, Corning, NY, United States).

### Cells and Cell Culture

MHCC97H cells, established at the Liver Cancer Institute of Fudan University, were grown in Dulbecco’s Modified Eagle’s Medium (DMEM, Gibco, United States) supplemented with 10% fetal bovine serum (FBS, Biowest, South America Origin) and 1% penicillin/streptomycin (Gibco, United States). Hep3B cells, obtained from the Cell Bank of Shanghai Institute of Biochemistry and Cell Biology (CAS), were cultured in Minimum Essential Medium (MEM, Gibco, Gibco, United States) with 12.5% FBS and 1% penicillin/streptomycin. Approximately 1 × 10^6^ HCC cells in a 0.3-ml culture medium were spread onto an FN-coated gel substrate and cultured for 2 h at room temperature. Subsequently, a 10-ml medium was added into a dish and the cells were transferred into the incubator for the next 48 h culture at 37°C with 5% CO^2^. Cells were collected from the gel surface with a cell scraper for the following analysis.

### Metformin

Metformin was purchased from Sigma-Aldrich (St. Louis, MO, Gibco, United States). Metformin was dissolved in 1 × PBS at a concentration of 1,000 mM to be a stock solution for storage.

### Cell Proliferation Assay

Cell viability was detected by cell counting kit-8 assay (CCK-8, DOJINDO, Kyushu, Japan). About 1 × 10^4^ HCC cells in 100 μl were transferred into a 96-well plate. When they grew and reached approximate 70% confluence, the cells were treated with different concentrations of metformin (0, 2, 4, 8, 16, 32, 64, 100 mM) for 24 h. Subsequently, the culture supernatant was abandoned and 10 μl CCK-8 reagent was added into the well for 2 h incubation at 37°C. Absorbance was measured by a spectrophotometer at 450-nm wavelength, and the absorbance of untreated cells was considered as 100% cell viability.

### Cell Motility and Invasion Assay

Cell motility and invasion abilities were examined using Boyden Chamber Assay (Corning, United States). Briefly, for cell motility assay, 200 μl HCC cells (1 × 10^6^/ml) pretreated with metformin were suspended in a serum-free medium and delivered into the upper chamber. A complete culture medium with 20% FBS was added into the lower chamber as a chemoattractant. After 36 hours of culture, the migrated cells at the bottom membrane were fixed by 4% paraformaldehyde and stained with crystal violet. The number of migrated cells in five random fields was counted under a light microscope. For cell invasion assay, the procedures were the same as that of migration assay except for the Matrigel-coated bottom membrane (Corning, NY, United States) of the upper chamber and 48 h cell culture.

### Viability Analysis of HCC Cells Grown on 16-kPa and 6-kPa Stiffness Substrates

HCC cells grown on 16- and 6-kPa stiffness substrates in a 12-well plate were divided into the metformin intervention group and the untreated group. The treated and untreated HCC cells were continuously imaged and recorded for 72 h using Cell-IQ continuous live cell imaging and analysis platform (Chip-Man, Finland). Dividing cells were automatically identified and counted by computer software “Cell-IQ Analyser,” and the number of dividing cells at different time periods represented the proliferative viability of the HCC cells.

### Migration of HCC Cells Grown on 16-kPa and 6-kPa Stiffness Substrates

HCC cells grown on 16- and 6-kPa stiffness substrates in a 12-well plate were divided into the metformin intervention group and the untreated group. The trace of individual cells in two groups was continuously imaged and recorded every one hour for 48 h by using Cell-IQ instrument described above. Individual cell tracking was performed by the “Manual tracking” plugin in ImageJ software, which enables the selection and tracking of a cell and its position in each frame. Acquired data was then analyzed by the “Chemotaxis tool” software to obtain cell migration speeds.

### Quantitative Reverse Transcription Polymerase Chain Reaction (qRT-PCR)

Total RNA was extracted from HCC cells using TRIzol reagent (Invitrogen, United States) and then reverse-transcribed into cDNA using First-Strand Synthesis Kit (Thermo Scientific, United States). The target gene was amplified in SYBR Green PCR Master Mix (Yeasen Biotech, China) with a specific primer by a QuantStudio^®^ 5 Real-Time PCR instrument (96-well 0.2-ml Block). The relative expression of target gene mRNA was normalized to GAPDH and calculated by using the 2^–ΔΔCt^ method. Three replicates were set for each gene, and the primer sequences of the detected genes are listed in Table 1.

**TABLE 1:** Primer sequence used for qRT-PCR.

Gene	Forward primer (5′–3′)	Reverse primer (5′–3′)
PTEN	GCTGGAAAGGGACGAACTGG	CTTGTCTTCCCGTCGTGTGG
MMP2	GTTCATTTGGCGGACTGT	AGGGTGCTGGCTGAGTAG
MMP9	GGGACGCAGACATCGTCATC	TCGTCATCGTCGAAATGGGC
GAPDH	CTCCTCCACCTTTGACGC	CCACCACCCTGTTGCTGT

### HCC Tissue Microarray and Immunohistochemistry

A HCC tissue microarray constructed previously was derived from buffalo rat HCC models with different liver stiffness backgrounds (13, 17). A tissue microarray was composed of six HCC tissues with normal liver stiffness background, six HCC tissues with medium liver stiffness background, and six HCC tissues with high liver stiffness background. Tissue microarrays were incubated with a primary antibody against PTEN (1:100, Abcam), MMP2 (1:50, Boster), MMP9 (1:50, Boster), respectively, at 4°C overnight and then reacted with biotin-labeled goat anti-rabbit IgG (SA1022, Boster). Photographs of the stained slides were captured by Aperio ImageScope v12.3.3, and the intensity of the stained area was measured and calculated by ImageJ software (United States). The detailed quantitative value is found in Supplementary Table S1.

### Western Blot

Total proteins of HCC cells were extracted in RIPA buffer (Beyotime, China) supplemented with 1 mM PMSF (Beyotime, China) and 10% PhosSTOP (Roche, Switzerland). Western blot was done as the method in our previous work (13). The primary antibodies were diluted as follows: GAPDH (1:1000, Cell Signal Technology), PTEN (1:1000, Cell Signal Technology), MMP2 (1:1000, Proteintech), MMP9 (1:1000, Cell Signal Technology), PI3K p110α (1:1000, Cell Signal Technology), Akt (1:000, Cell Signal Technology), p-Akt Thr308 (1:000, Cell Signal Technology), and integrin β1 (1:1000, Cell Signal Technology). The HRP-conjugated secondary antibody (goat anti-mouse or goat anti-rabbit IgG, Proteintech) was diluted as 1:5000. The target protein band was visualized using an electrochemiluminescence kit (Tanon, China). The density of the protein band was quantified by ImageJ software (United States).

### Establishment of HCC Cells With LV-shRNA-ITGβ1 Using Lentiviral-Mediated RNAi Technology

The interference sequence of human integrin β1 (ITGβ1, 5′-CCTCCAGATGACATAGAAA-3′) was designed and cloned into the plasmid pGCSIL. Subsequently, 20 μg recombinant plasmid, 15 μg pHelper 1.0 plasmid, and 10 μg pHelper 2.0 plasmid were incubated in Opti-MEM for 5 min at room temperature. The mixture was co-transfected into HEK293T cells by Lipofectamine 2000. The viral supernatant was harvested, and the viral titer was determined. Design of interference sequence, lentivirus preparations, and packaging were collaborated with Shanghai GeneChem, Co. Ltd, China. HCC cells were infected with LV-shRNA-ITGβ1 plus 1 × Hitrans GP (MHCC97H cells with a multiplicity of infection (MOI) of 5, Hep3B cells with a multiplicity of infection (MOI) of 10) to obtain stably transfected HCC cells with LV-shRNA-ITGβ1.

### PI3K Inhibition Assay

LY294002 (Cell Signal Technology), a selective PI3K inhibitor, inhibits phosphorylation of downstream molecule Akt (Thr308/Ser473). LY294002 was dissolved in 488 μl DMSO to reach 10 mM storage concentration and then diluted into 20 μM work solution in the complete culture medium for cell intervention. An equal amount of DMSO was set as the control (V_*DMSO*_:V_*complete culture medium*_ = 2‰). HCC cells grown on high-stiffness substrates were treated with LY294002 for 48 h before they were collected from gels.

### Dual Luciferase Reporter Assay

The wild type of PTEN-3′UTR containing the binding site of miR-17-5p and its mutant type were recombined into pmirGLO vectors (Bio-link, China) respectively. 293T cells in a good growth state were cultured into a 24-well culture plate on the day before plasmid transfection. Plasmid transfection experiments were carried out based on the designed groups in the experiment. The expression of fluorescent marker genes in cells was measured under a fluorescence microscope after 24 h of transfection, and luciferase expression was assayed with the Dual-Luciferase^®^ Reporter Assay System Kit (E1910, Promega).

### MicroRNA RT-PCR

Total RNA was extracted the same as the method described above, and the cDNA of miRNA was reverse-transcribed using the reverse translate kit (GeneCopoeia, Guangzhou, China) according to the manufacturer’s protocol. Sequence-specific primers for U6-2 and miR-17-5p were synthesized from GeneCopoeia. The relative expression level of miR-17-5p in each sample was calculated using the 2^–ΔΔct^ method. hsnRNA U6-2 was selected as an endogenous reference. All experiments were performed independently in triplicate.

### MicroRNA-17-5p Transfection

MiR-17-5p mimic, mimic-NC, miR-17-5p inhibitor, and inhibitor-NC were designed and synthesized by Sangon Biotech (Shanghai, China) with following sequences: hsa-miR-17-5p mimic: F: 5′-CAAAGUGCUUACAGUGCAGGUAG-3′, R: 5′-CUACCUGCACUGUAAGCACUUUG-3′; mimic-NC: F: 5′-UUCUCCGAACGUGUCACGUTT-3′, R: 5′-ACGUGACACGUUCGGAGAATT-3′; hsa-miR-17-5p inhibitor: 5′-CUACCUGCACUGUAAGCACUUUG-3′; hsa-miR-17-5p inhibitor-NC: 5′-CAGUACUUUUGUGUAGUACAA-3′. Hsa-miR-17-5p mimic (50 nmol/l) was transfected into HCC cells grown on a lower-stiffness substrate using Lipofectamine 2000 (Invitrogen, United States). Hsa-miR-17-5p mimic-NC was set as the control. Contrarily, hsa-miR-17-5p inhibitor (50 nmol/l) was transfected into HCC cells grown on a higher-stiffness substrate and hsa-miR-17-5p inhibitor-NC also acted as the control. Transient transfections were performed according to manufacturers’ instructions.

### Statistical Analysis

GraphPad Prism 8.0 (San Diego, CA, United States) and SPSS 22.0 statistical software (SPSS Inc., Chicago, IL, United States) were used for all statistical analyses. Data were presented as mean ± standard error of the mean (SEM). Statistical analysis was performed by Student’s *t*-test. *p* < 0.05 is considered to be statistically significant.

## Results

### Metformin Inhibits Proliferation, Migration, and Invasion of HCC Cells

**FIGURE 1:**
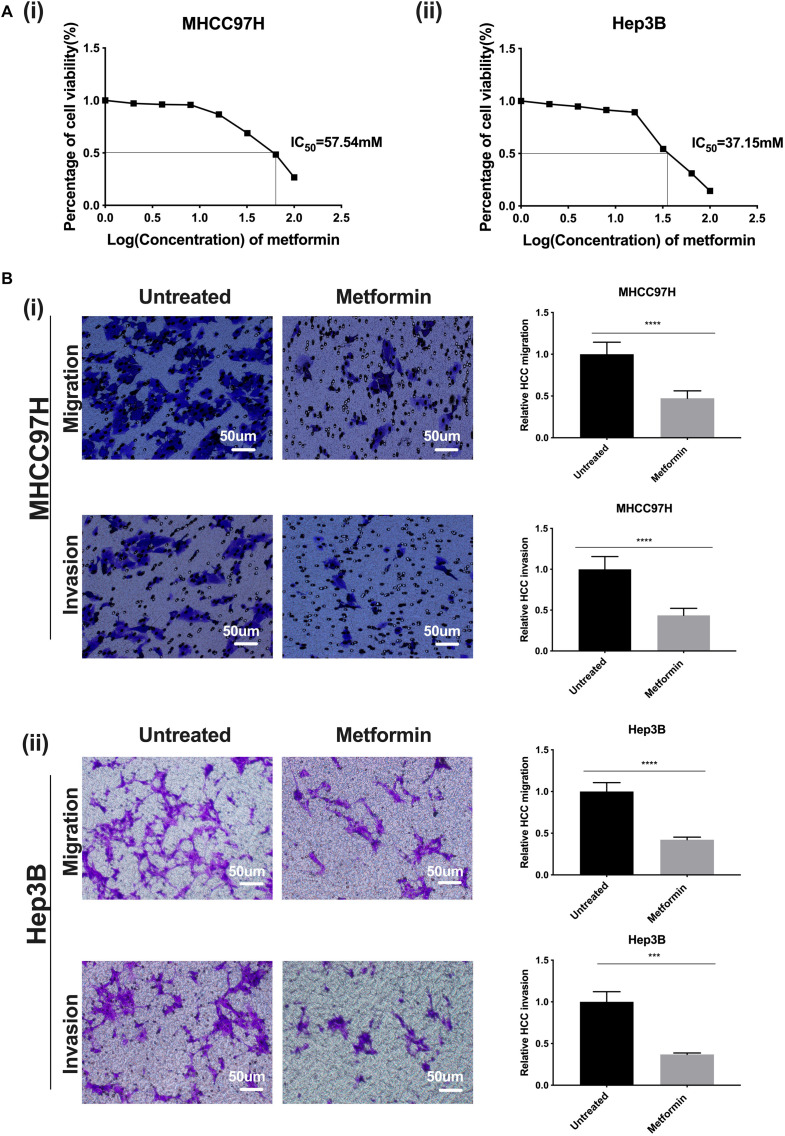
Metformin inhibits proliferation, migration, and invasion of HCC cells. **(A) (i)** A dose–response curve of metformin in MHCC97H cells in concentrations of 0, 2, 4, 8, 16, 32, 64, and 100 mM for 24 h. **(ii)** A dose–response curve of metformin in Hep3B cells in concentrations of 0, 2, 4, 8, 16, 32, 64, and 100 mM for 24 h. **(B) (i)** The migration and invasion abilities of MHCC97H cells were remarkably inhibited after metformin treatment (27 mM) for 36 or 48 h. **(ii)** The migration and invasion abilities of Hep3B cells were remarkably inhibited after metformin treatment (22 mM) for 36 or 48 h. Error bar represents a standard error of the means (SEM), *p < 0.05, **p < 0.01, ***p < 0.001, and ****p < 0.0001.

Based on a dose–response curve of metformin in HCC cells, we determined the half inhibition concentration (IC50) of metformin as 57.54 mM in MHCC97H cells and 37.15 mM in Hep3B cells (Figure 1A i, ii). Afterward, we defined the most suitable intervention concentration of metformin as 27 mM in MHCC97H cells and 22 mM in Hep3B cells, which corresponded to the 80% survival rate of HCC cells, for the following experiments. The viability of HCC cells treated with metformin was significantly suppressed compared with that of the untreated cells; moreover, the inhibition effect of metformin was dose-dependent, indicating that metformin possesses better inhibition effects on the proliferation and growth of HCC cells. Simultaneously, cell motility and invasion assay showed that the number of the migrated or invaded HCC cells in exposure to metformin were all obviously decreased compared with that of the untreated HCC cells, demonstrating that metformin is able to markedly weaken the migratory and invasive ability of HCC cells (Figure 1B i, ii).

### Higher Matrix Stiffness Attenuates the Inhibition Effects of Metformin on HCC Invasion and Metastasis

**FIGURE 2:**
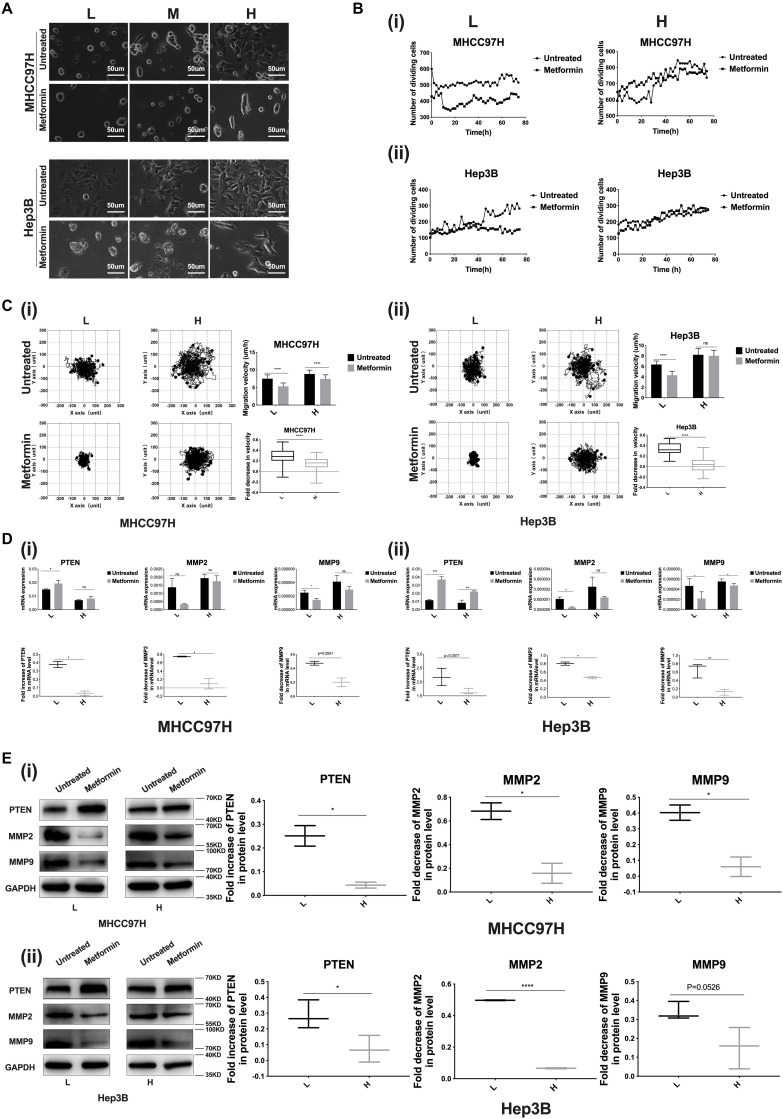
Higher matrix stiffness resists the inhibition effects of metformin on HCC invasion and metastasis. **(A)** Morphology of HCC cells grown on low (L, 6 kPa), median (M, 10 kPa), and high (H, 16 kPa) stiffness substrates before and after metformin intervention (27 mM for MHCC97H cells and 22 mM for Hep3B cells for 24 h). **(B) (i)** Proliferation viability of MHCC97H cells grown on 16 and 6-kPa stiffness substrates before and after 27-mM metformin intervention for 72 h. **(ii)** Proliferation viability of Hep3B cells grown on 16-kPa and 6-kPa stiffness substrates before and after 22-mM metformin intervention for 72 h. **(C) (i)** Migration ability changes of MHCC97H cells grown on 16 and 6-kPa stiffness substrates after 27-mM metformin intervention for 48 h. **(ii)** Migration ability changes of Hep3B cells grown on 16 and 6-kPa stiffness substrates after 22-mM metformin intervention for 48 h. **(D) (i)** mRNA expression changes of PTEN, MMP2, and MMP9 in MHCC97H cells under higher or lower stiffness stimulation after 27-mM metformin intervention for 24 h. **(ii)** mRNA expression changes of PTEN, MMP2, and MMP9 in Hep3B cells under higher or lower stiffness stimulation after 22-mM metformin intervention for 24 h. **(E) (i)** Protein expression changes of PTEN, MMP2, and MMP9 in MHCC97H cells after 27-mM metformin intervention for 24 h under higher or lower stiffness stimulation. **(ii)** Protein expression changes of PTEN, MMP2, and MMP9 in Hep3B cells after 22-mM metformin intervention for 24 h under higher or lower stiffness stimulation. Error bar represents SEM, *p < 0.05, **p < 0.01, ***p < 0.001, and ****p < 0.0001.

We established 6-, 10-, and 16-kPa stiffness substrates as previously reported (13), which mirrored the stiffness level of normal, fibrotic, and cirrhotic livers, respectively, to explore matrix stiffness-mediated metformin resistance in HCC cells. Considering that morphological alteration of cancer cells frequently reflects the changes of their malignant biological behaviors, we firstly observed the morphology of HCC cells grown on different-stiffness substrates and found that HCC cells presented a more extended and expanded morphology as matrix stiffness increased; simultaneously, we mentioned that little change occurred in the shape and spread area of HCC cells grown on the 16-kPa stiffness substrate in response to metformin compared to the cells on 10- and 6-kPa stiffness substrates (Figure 2A), implying that the higher-stiffness substrate may resist the effects of metformin on cell morphology such as shape and spread area. Subsequently, we further analyzed whether increased matrix stiffness influenced the inhibition effects of metformin on proliferation, migration, invasion and metastasis. As shown in Figure 2B i, ii, the division activity of HCC cells grown on the higher-stiffness substrate was stronger, and the inhibition of proliferation after metformin intervention was weaker than those on the lower-stiffness substrate, suggesting that higher matrix stiffness contributes to promoting cell proliferation and resists metformin inhibitory efficacy in proliferation.

In the presence or absence of metformin, HCC cells grown on the higher-stiffness substrate migrated faster than those on the lower-stiffness substrate, in agreement with the results of our previous study (17). Besides, metformin intervention suppressed the migratory ability of HCC cells irrespective of being grown on the lower- or higher-stiffness substrate, but the reduction of cell velocity after metformin intervention was more significant for HCC cells cultured on the 6-kPa stiffness substrate compared to the cells on the 16-kPa stiffness substrate (Figure 2C i, ii), suggesting that higher matrix stiffness counteracts the inhibition effect of metformin on HCC cell migration.

We further detected the expressions of invasive and metastasis-associated genes (PTEN, MMP2, and MMP9) in HCC cells grown on 6- and 16-kPa stiffness substrates in the presence or absence of metformin intervention and found that metformin intervention could remarkably upregulate the expression of PTEN and downregulate the expressions of MMP2 and MMP9 in HCC cells irrespective of being grown on a 16- or 6-kPa stiffness substrate. More importantly, our results also showed that higher matrix stiffness attenuated the effects of metformin on PTEN, MMP9, and MMP2 expressions obviously (Figures 2D,E), supporting our hypothesis by which matrix stiffness participates in inducing metformin resistance. Taken together, higher matrix stiffness weakened the intervention effects of metformin on proliferation, invasion and metastasis.

### Increased Matrix Stiffness Activates the PI3K/Akt Signal Pathway, Simultaneously Upregulates MMP2 and MMP9 Expressions, and Downregulates PTEN Expression

**FIGURE 3:**
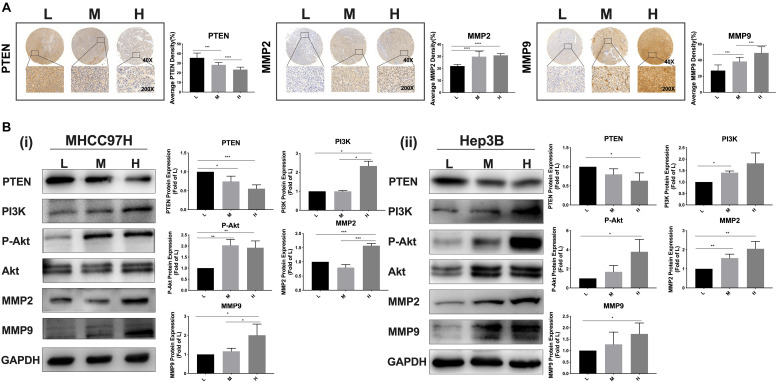
Increased matrix stiffness activates the PI3K/Akt signal pathway, simultaneously upregulates MMP2/MMP9 expressions, and downregulates PTEN expression. **(A)** The expression levels of PTEN, MMP2, and MMP9 in HCC tissues derived from buffalo rat HCC models with different liver stiffness backgrounds. L, normal liver stiffness group; M, medium liver stiffness group; H, high liver stiffness group. **(B)** Increased matrix stiffness activated the PI3K/Akt/MMP signaling pathway, simultaneously upregulated MMP2/MMP9 expressions, and downregulated the PTEN expression in MHCC97H and Hep3B cells. Error bar represents SEM, *p < 0.05, **p < 0.01, ***p < 0.001, and ****p < 0.0001.

Given that metformin intervention remarkably altered the expression levels of PTEN, MMP2, and MMP9 in HCC cells, we further clarified whether matrix stiffness also modulated the expressions of these invasive/metastasis-associated molecules. Our results demonstrated that increased matrix stiffness not only upregulated MMP2 and MMP9 expressions in HCC cells but also downregulated PTEN expression, which is contrary to the results of metformin intervention. Subsequently, analysis of an HCC tissue microarray derived from buffalo rat HCC models with different liver stiffness backgrounds (13) also revealed that higher liver stiffness backgrounds promoted MMP2 and MMP9 expression but suppressed PTEN expression in rat HCC tissues (Figure 3A), in agreement with the results of experiments *in vitro*. Thereby, PTEN, MMP2, and MMP9 may be the common targets in HCC cells for matrix stiffness stimulation and metformin intervention. PTEN acts as a negative regulator of PI3K signaling, whose main substrate is phosphatidylinositol-3,4,5-triphosphate (PIP3) (19, 20). Moreover, the activation of the PI3K/Akt signaling pathway is positively correlated with the expressions of MMP2 and MMP9 (21, 22). As a result, we continued to analyze the activation of the PI3K/Akt signaling pathway in HCC cells grown on different-stiffness substrates and found that increased matrix stiffness evidently activated the PI3K/Akt signaling pathway (Figure 3B), illustrating that a common pathway of PTEN/PI3K/Akt/MMPs activated by the mechanical stiffness signal and inactivated by metformin intervention may contribute to matrix stiffness-caused metformin resistance.

### A Common Signaling Pathway of PTEN/PI3K/Akt/MMPs Activated by Mechanical Stiffness Signal and Inactivated by Metformin Contributes to Matrix Stiffness-Caused Metformin Resistance

**FIGURE 4:**
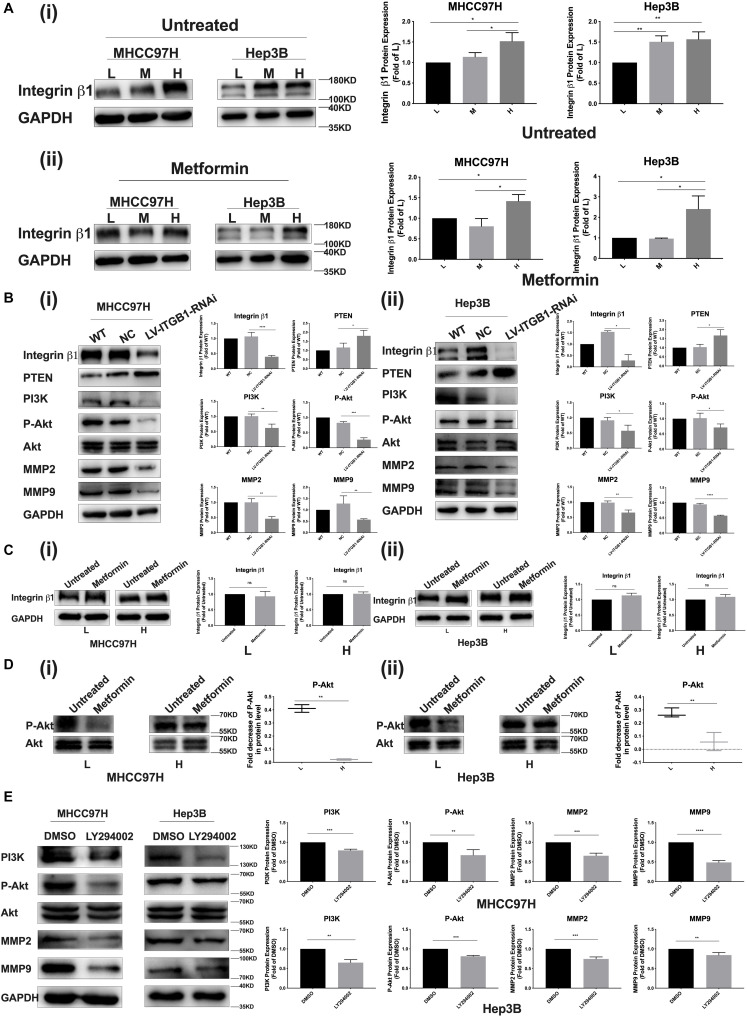
PTEN/PI3K/Akt/MMPs as a common signaling pathway contributes to matrix stiffness-caused metformin resistance. **(A) (i,ii)** Irrespective of metformin intervention or not, integrin β1 expression shows an obvious increase tendency in two HCC cells as matrix stiffness increases. **(B) (i,ii)** Knockdown of integrin β1 significantly upregulates the expressions of PTEN and partially inhibited the activation of the PI3K/Akt pathway in HCC cells grown on 16-kPa stiffness substrate. **(C) (i,ii)** Metformin intervention (27 mM for MHCC97H cells and 22 mM for Hep3B cells) had little effect on the expression of integrin β1 in HCC cells grown on 16 or 6-kPa stiffness substrates. **(D) (i)** MHCC97H cells grown on lower-stiffness substrate exhibited an obvious decrease in Akt phosphorylation after 27-mM metformin treatment for 24 h compared with the cells grown on a higher-stiffness substrate. **(ii)** Hep3B cells grown on a lower-stiffness substrate exhibited an obvious decrease in Akt phosphorylation after 22-mM metformin treatment for 24 h compared with the cells grown on higher-stiffness substrate. **(E)** PI3K inhibitor (LY294002, 20 μM) suppressed Akt phosphorylation and MMP2/MMP9 expression in HCC cells grown on the 16-kPa stiffness substrate. Error bar represents SEM, *p < 0.05, **p < 0.01, ***p < 0.001, and ****p < 0.0001.

Integrin β1, as a stiffness sensor molecule identified previously, delivers extracellular matrix (ECM) mechanical signals into HCC cells and initiate downstream cascade molecular events (13). Here, as matrix stiffness increased, integrin β1 expression showed an increase tendency in HCC cells irrespective of metformin treatment or not (Figure 4A i, ii). Simultaneously, integrin β1 knockdown partially reversed PTEN expression and the activation of the PI3K/Akt/MMPs pathway in HCC cells grown on the 16-kPa stiffness substrate (Figure 4B). These results not only reconfirmed that integrin β1 acted as a stiffness sensor molecule to transduce stiffness the mechanical signal into HCC cells but also further validated that stiffness mechanical signal activated the PTEN/PI3K/Akt pathway to upregulate MMP9 and MMP2 expressions. After metformin intervention, HCC cells grown on different-stiffness substrates had no significant change in expression of integrin β1 (Figure 4C i, ii) but exhibited a more obvious decrease in Akt phosphorylation in HCC cells grown on a lower-stiffness substrate compared with the cells grown on a higher-stiffness substrate (Figure 4D i, ii). These findings suggest that metformin influences the activation of the PI3K/Akt pathway and PTEN expression but has no effect on integrin β1 expression. We further used PI3K inhibitor LY294002 to examine the phosphorylation level of Akt and MMP2/MMP9 expressions in HCC cells grown on a high-stiffness substrate; the PI3K inhibitor significantly suppressed the level of Akt phosphorylation and downregulated the expressions of MMP2 and MMP9, indicating that the PI3K/Akt pathway specifically regulates MMP2 and MMP9 expression in HCC cells (Figure 4E). Taken altogether, a common pathway of PTEN/PI3K/Akt/MMPs activated by mechanical stiffness signal and inactivated by metformin contributes to matrix stiffness-caused metformin resistance.

### MicroRNA-17-5p Participates in Stiffness-Mediated Effect on PTEN Expression

**FIGURE 5:**
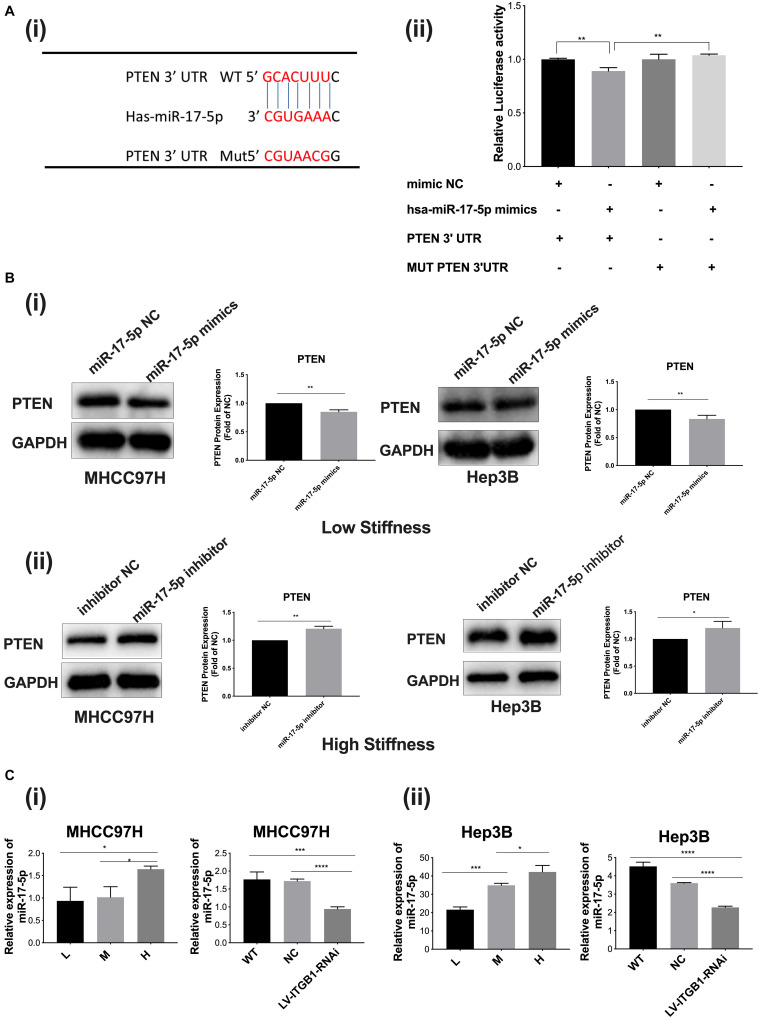
MiR-17-5p participates in stiffness-mediated effect on PTEN expression. **(A) (i)** The predicted binding sites of miR-17-5p in the PTEN mRNA 3′-UTR using the TargetScan database and the mutation sequences of PTEN mRNA 3′-UTR. **(ii)** Dual-luciferase reporter assay validated that miR-17-5p specifically binds 3′-UTR of PTEN. **(B) (i,ii)** PTEN expression is significantly decreased in HCC cells with miR-17-5p mimics (50 nmol/l) cultured on the 6-kPa stiffness substrate and increased in HCC cells with miR-17-5p inhibitor (50 nmol/l) under the 16-kPa stiffness substrate. **(C) (i,ii)** Higher matrix stiffness upregulates the expression of miR-17-5p, and knockdown of integrin β1 obviously reverses this change. Error bar represents SEM, *p < 0.05, **p < 0.01, ***p < 0.001, and ****p < 0.0001.

We further explored whether the upstream microRNA also participated in the stiffness-mediated effect on PTEN expression. Using the TargetScan database (Human 7.2, United States), we predicted that miR-17-5p had a possible binding site in the PTEN mRNA 3′-UTR, suggesting that miR-17-5p may be a potential microRNA to regulate PTEN expression (Figure 5A i). Dual-luciferase reporter assay validated that miR-17-5p specifically bound 3′-UTR of PTEN, indicating that miR-17-5p may reversely regulate PTEN expression (Figure 5A ii). Subsequently, we transfected miR-17-5p mimics or miR-17-5p inhibitor into HCC cells to upregulate or downregulate miR-17-5p and detected its roles in the expression of target protein PTEN. The results demonstrated that PTEN expression was significantly decreased in the group with miR-17-5p mimics and increased in the group with the miR-17-5p inhibitor (Figure 5B i, ii), all revealing the regulation role of miR-17-5p in PTEN expression. MiR-17-5p belongs to the miRNA-17-92 cluster, which are involved in biological development of various cancers (23). Since higher stiffness as a physical factor promoted malignant features of HCC, we wondered whether miR-17-5p participates in stiffness-mediated effect on PTEN expression. As shown in Figure 5C i, ii, with increase in matrix stiffness, the relative expression of miR-17-5p showed an increase tendency, which is opposite to PTEN expression described above. Integrin β1 knockdown weakened stiffness signal stimulation and partially reversed miR-17-5p expression in HCC cells grown on the 16-kPa stiffness substrate. All the above results indicated that microRNA-17-5p participated in stiffness-mediated effect on PTEN expression.

## Discussion

Biochemical cue-regulated drug resistance and tolerance have been well documented (6, 7, 10). However, how biomechanical cues within the tumor microenvironment influence or modulate therapeutic resistance remains largely uncharacterized. Here we devoted to clarify the potential roles of biophysical mechanical signals in metformin intervention resistance on HCC invasion and metastasis and discussed its underlying mechanism. Clinical data have demonstrated that most of HCC patients develop on a background of fibrotic or cirrhotic liver, and HCC patients with advanced cirrhotic liver have lower medium survival time and unfavorable outcome (24). Liver stiffness acts as an indicator in clinic to assess the risk of HCC development and progression (25, 26). In addition to clinical evidences, some basic researches also support that increased matrix stiffness heightens malignant characteristics of cancer including proliferation, invasion, metastasis, angiogenesis, and recurrence (13, 27, 28). In addition, increased matrix stiffness exerts significant roles in chemotherapeutic resistance to cisplatin, 5-FU, and paclitaxel in HCC (15, 18). In our previous research, HCC cells grown on higher-stiffness substrate also exhibit resistance to oxaliplatin-induced apoptosis (12). All these studies implicate that there probably exists a close linkage between matrix stiffness and therapeutic resistance.

Metformin, as a well-tolerated traditional first-line anti-hyperglycemic drug for diabetes, has recently been found to have therapeutic effects in obesity, non-alcoholic fatty liver disease (NAFLD), polycystic ovary syndrome (PCOS), and metabolic syndrome (29, 30). Besides, metformin alone or combined with other chemotherapy drugs exhibits an obvious inhibition effect on the malignant properties of various cancers including HCC (3, 5, 31, 32). Our data also revealed that metformin at appropriate concentrations obviously suppressed proliferation, motility, invasion and metastasis of HCC cells. However, little is known about the underlying mechanisms of metformin resistance and tolerance on HCC. Accordingly, we speculated that higher matrix stiffness might attenuate the inhibition effects of metformin on HCC invasion and metastasis.

The expression levels of PTEN, MMP2, and MMP9 often indicate invasive and metastatic ability of tumor cells (33–35). Metformin alone or in combination with curcumin presents a better intervention effect on HCC invasion and metastasis via upregulating PTEN expression and downregulating MMP2 and MMP9 expressions (5). Our results also demonstrated that metformin was able to markedly weaken the proliferation, migratory, and invasive abilities of HCC cells, supporting antitumor roles of metformin in HCC. Simultaneously, we obtained another important finding that increased matrix stiffness significantly attenuated the intervention effects of metformin on proliferation, migration, invasion, and metastasis. These results confirmed our hypothesis and laid a solid foundation for following mechanism exploration.

PTEN negatively mediates the PI3K/Akt signaling pathway (20), and the activation of the PI3K/Akt pathway is positively correlated with the expressions of MMP2 and MMP9 (21, 22). Our data suggested that higher matrix stiffness stimulation reduced metformin-induced PTEN upregulation and MMP2/MMP9 downregulation. Because activation of the PI3K/Akt signaling pathway is associated with PTEN and MMP2/MMP9 expression reported in previous studies, we further inferred that increased matrix stiffness might downregulate PTEN to activate the PI3K/Akt signaling pathway and subsequently promote MMP2/MMP9 expression. The results *in vitro* and *in vivo* confirmed that increased matrix stiffness remarkably suppressed PTEN expression and improved MMP2 and MMP9 expressions in HCC cells. Meanwhile, increased matrix stiffness also activated the PI3K/Akt pathway. Since metformin and matrix stiffness have opposite effects on PTEN expression, PTEN as an upstream molecule, which could negatively regulate the PI3K/Akt/MMP signaling pathway, is supposed to be a common target of matrix stiffness and metformin. Integrin β1 was identified previously as stiffness sensor molecule, which mediates the transmission of ECM stiffness signals into HCC cells (13, 15, 36). Here, knockdown of integrin β1 upregulated PTEN expression in HCC cells grown on higher-stiffness substrate and also inhibited the activation of the PI3K/Akt/MMP pathway. Additionally, the PI3K inhibitor specifically inactivated the PI3K/Akt pathway and downregulated MMP2 and MMP9 expression in HCC cells. All these data suggested that increased matrix stiffness counteracted the intervention effects of metformin through the PTEN/PI3K/Akt/MMP pathway.

MicroRNAs could contribute to cell–matrix interactions and promote integrin-dependent cell adhesion (37). Also, miRNAs may regulate biological behaviors of cancer cells in response to mechanical signal and ECM stiffness. A study shows that the expressions of 122 microRNAs encoding cytoskeletal, contractile, adhesive, and ECM proteins are elevated in endothelial cells grown on stiffer substrates (38). Another study suggests that mechanics modulate miR-18a-dependent PTEN expression to control malignant progression of breast cancer (39). So, we continued to elucidate whether the upstream microRNA participated in the stiffness-mediated effect on PTEN expression. Our results revealed that higher matrix stiffness obviously upregulated miR-17-5p expression and miR-17-5p negatively regulated downstream target molecule PTEN. Dual-luciferase reporter assay and miR-17-5p mimic or inhibitor transfection analysis all supported that miR-17-5p participated in the stiffness-mediated effect on PTEN expression, which were consistent with the results of previous study (40). Therefore, higher matrix stiffness-upregulated miR-17-5p, which negatively mediates PTEN, triggered a cascade of the downstream signaling pathway.

Although all the data *in vitro* support high matrix stiffness, resisting the inhibition effect of metformin on HCC invasion and metastasis, matrix stiffness-caused metformin resistance still deserves to be further validated in HCC animal models in the future.

## Conclusion

**FIGURE 6:**
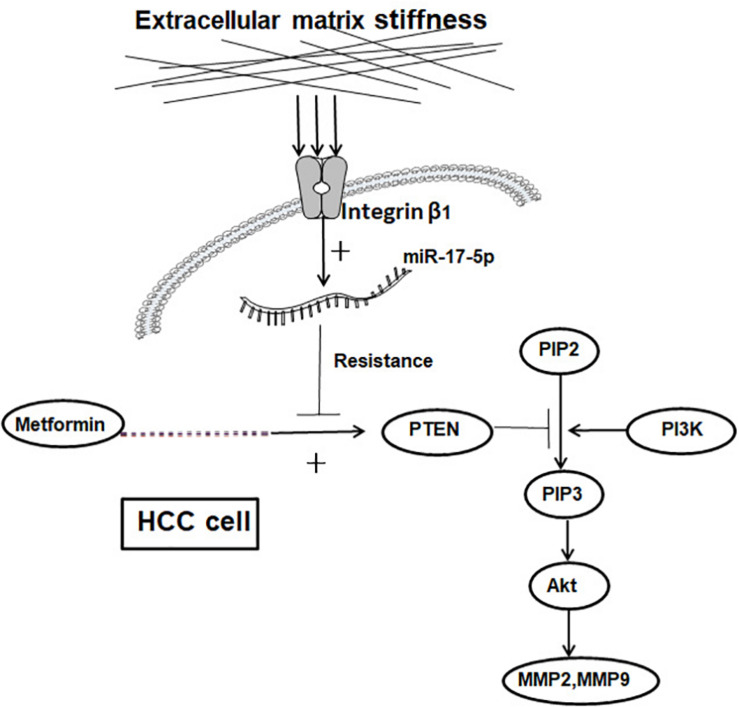
Schematic diagram of the proposed molecular mechanism by which higher matrix stiffness attenuates the inhibitory effect of metformin on HCC invasion and metastasis.

Higher matrix stiffness attenuates a metformin-induced inhibitory effect on HCC invasion and metastasis, and a common pathway of PTEN/PI3K/Akt/MMPs, which was activated by mechanical stiffness signal and inactivated by metformin, contributing to matrix stiffness-caused metformin resistance (Figure 6). To the best of our knowledge, this is the first report to clarify the mechanism of metformin intervention resistance from the perspective of the tumor biophysical microenvironment, maybe providing new directions and ideas for prevention of therapeutic resistance in HCC.

## Data Availability Statement

The original contributions presented in the study are included in the article/Supplementary Material, further inquiries can be directed to the corresponding authors.

## Author Contributions

YY and JC conceived and designed the study. XG performed most of the experiments and wrote the manuscript. XQ and XX helped culture the cells. JH, JQ, and YW analyzed the data. YZ, XZ, and ML contributed to language polishing and modification. All authors read and approved the final manuscript.

## Conflict of Interest

The authors declare that the research was conducted in the absence of any commercial or financial relationships that could be construed as a potential conflict of interest.

## Supplementary Material

FIGURE S1 Schematic diagram of metformin intervention on HCC cells grown on different-stiffness substrates.

FIGURE S2 Assessment of miRNA-17-5p transfection efficiency. **(A)** MiRNA-17-5p mimic (50 nmol/l) transient transfection in MHCC97H and Hep3B cells. **(B)** MiRNA-17-5p inhibitor (50 nmol/l) transient transfection in MHCC97H and Hep3B cells.

FIGURE S3 Metformin has little effect on miR-17-5p expression level in HCC cells (27 mM for MHCC97H cells and 22 mM for Hep3B cells for 24 h).

TABLE S1 Quantitative density area value of immunohistochemistry in HCC tissue microarray.

## References

[1] Tseng CH. Metformin and risk of hepatocellular carcinoma in patients with type 2 diabetes. *Liver Int.* (2018) 38:2018–27. 10.1111/liv.13872 29956875

[2] Vacante F Senesi P Montesano A Paini S Luzi L Terruzzi I. Metformin counteracts HCC progression and metastasis enhancing KLF6/p21 expression and downregulating the IGF axis. *Int J Endocrinol.* (2019) 2019:7570146. 10.1155/2019/7570146 30774659 PMC6350585

[3] Hu L Zeng Z Xia Q Liu Z Feng X Chen J Metformin attenuates hepatoma cell proliferation by decreasing glycolytic flux through the HIF-1alpha/PFKFB3/PFK1 pathway. *Life Sci.* (2019) 239:116966. 10.1016/j.lfs.2019.116966 31626790

[4] Shankaraiah RC Callegari E Guerriero P Rimessi A Pinton P Gramantieri L Metformin prevents liver tumourigenesis by attenuating fibrosis in a transgenic mouse model of hepatocellular carcinoma. *Oncogene.* (2019) 38:7035–45. 10.1038/s41388-019-0942-z 31409896

[5] Zhang HH Zhang Y Cheng YN Gong FL Cao ZQ Yu LG Metformin incombination with curcumin inhibits the growth, metastasis, and angiogenesis of hepatocellular carcinoma in vitro and in vivo. *Mol Carcinogen.* (2018) 57:44–56. 10.1002/mc.22718 28833603

[6] Russo M Crisafulli G Sogari A Reilly NM Arena S Lamba S Adaptive mutability of colorectal cancers in response to targeted therapies. *Science (New York N Y).* (2019) 366:eaav4474. 10.1126/science.aav4474 31699882

[7] Misale S Yaeger R Hobor S Scala E Janakiraman M Liska D Emergence of KRAS mutations and acquired resistance to anti-EGFR therapy in colorectal cancer. *Nature.* (2012) 486:532–6. 10.1038/nature11156 22722830 PMC3927413

[8] Diaz LA Jr. Williams RT Wu J Kinde I Hecht JR Berlin J The molecular evolution of acquired resistance to targeted EGFR blockade in colorectal cancers. *Nature.* (2012) 486:537–40. 10.1038/nature11219 22722843 PMC3436069

[9] Montero J Gstalder C Kim DJ Sadowicz D Miles W Manos M Destabilization of NOXA mRNA as a common resistance mechanism to targeted therapies. *Nat Commun.* (2019) 10:5157. 10.1038/s41467-019-12477-y 31727958 PMC6856172

[10] Morandi A Indraccolo S. Linking metabolic reprogramming to therapy resistance in cancer. *Biochim Biophys Acta Rev Cancer.* (2017) 1868:1–6. 10.1016/j.bbcan.2016.12.004 28065746

[11] Hirpara J Eu JQ Tan JKM Wong AL Clement MV Kong LR Metabolic reprogramming of oncogene-addicted cancer cells to OXPHOS as a mechanism of drug resistance. *Redox Biol.* (2019) 25:101076. 10.1016/j.redox.2018.101076 30642723 PMC6859574

[12] You Y Zheng Q Dong Y Xie X Wang Y Wu S Matrix stiffness-mediated effects on stemness characteristics occurring in HCC cells. *Oncotarget.* (2016) 7:32221–31. 10.18632/oncotarget.8515 27050147 PMC5078009

[13] Dong Y Xie X Wang Z Hu C Zheng Q Wang Y Increasing matrix stiffness upregulates vascular endothelial growth factor expression in hepatocellular carcinoma cells mediated by integrin beta1. *Biochem Biophys Res Commun.* (2014) 444:427–32. 10.1016/j.bbrc.2014.01.079 24472554

[14] Zhao G Cui J Qin Q Zhang J Liu L Deng S Mechanical stiffness of liver tissues in relation to integrin beta1 expression may influence the development of hepatic cirrhosis and hepatocellular carcinoma. *J Surg Oncol.* (2010) 102:482–9. 10.1002/jso.21613 20872952

[15] Schrader J Gordon-Walker TT Aucott RL van Deemter M Quaas A Walsh S Matrix stiffness modulates proliferation, chemotherapeutic response, and dormancy in hepatocellular carcinoma cells. *Hepatology.* (2011) 53:1192–205. 10.1002/hep.24108 21442631 PMC3076070

[16] Wu S Zheng Q Xing X Dong Y Wang Y You Y Matrix stiffness-upregulated LOXL2 promotes fibronectin production, MMP9 and CXCL12 expression and BMDCs recruitment to assist pre-metastatic niche formation. *J Exp Clin Cancer Res CR.* (2018) 37:99. 10.1186/s13046-018-0761-z 29728125 PMC5935912

[17] Dong Y Zheng Q Wang Z Lin X You Y Wu S Higher matrix stiffness as an independent initiator triggers epithelial-mesenchymal transition and facilitates HCC metastasis. *J Hematol Oncol.* (2019) 12:112. 10.1186/s13045-019-0795-5 31703598 PMC6839087

[18] Liu C Liu Y Xie HG Zhao S Xu XX Fan LX Role of three-dimensional matrix stiffness in regulating the chemoresistance of hepatocellular carcinoma cells. *Biotechnol Appl Biochem.* (2015) 62:556–62. 10.1002/bab.1302 25274163

[19] Carnero A Blanco-Aparicio C Renner O Link W Leal JF. The PTEN/PI3K/AKT signalling pathway in cancer, therapeutic implications. *Curr Cancer Drug Targets.* (2008) 8:187–98. 18473732 10.2174/156800908784293659

[20] Salmena L Carracedo A Pandolfi PP. Tenets of PTEN tumor suppression. *Cell.* (2008) 133:403–14. 10.1016/j.cell.2008.04.013 18455982

[21] Jiang WG Sanders AJ Katoh M Ungefroren H Gieseler F Prince M Tissue invasion and metastasis: molecular, biological and clinical perspectives. *Sem Cancer Biol.* (2015) 35:S244–75. 10.1016/j.semcancer.2015.03.008 25865774

[22] Liu Y Liu C Tan T Li S Tang S Chen X. Sinomenine sensitizes human gastric cancer cells to cisplatin through negative regulation of PI3K/AKT/Wnt signaling pathway. *Anti Cancer Drugs.* (2019) 30:983–90. 10.1097/cad.0000000000000834 31609766 PMC6824511

[23] Concepcion CP Bonetti C Ventura A. The microRNA-17-92 family of microRNA clusters in development and disease. *Cancer J (Sudbury Mass).* (2012) 18:262–7. 10.1097/PPO.0b013e318258b60a 22647363 PMC3592780

[24] Fattovich G Stroffolini T Zagni I Donato F. Hepatocellular carcinoma in cirrhosis: incidence and risk factors. *Gastroenterology.* (2004) 127(Suppl. 1):S35–50. 15508101 10.1053/j.gastro.2004.09.014

[25] Masuzaki R Tateishi R Yoshida H Goto E Sato T Ohki T Prospective risk assessment for hepatocellular carcinoma development in patients with chronic hepatitis C by transient elastography. *Hepatology.* (2009) 49:1954–61. 10.1002/hep.22870 19434742

[26] Masuzaki R Tateishi R Yoshida H Yoshida H Sato S Kato N Risk assessment of hepatocellular carcinoma in chronic hepatitis C patients by transient elastography. *J Clin Gastroenterol.* (2008) 42:839–43. 10.1097/mcg.0b013e318050074f 18668703

[27] Zhang R Ma M Dong G Yao RR Li JH Zheng QD Increased matrix stiffness promotes tumor progression of residual hepatocellular carcinoma after insufficient heat treatment. *Cancer Sci.* (2017) 108:1778–86. 10.1111/cas.13322 28699238 PMC5581508

[28] Chaudhuri O Koshy ST Branco da Cunha C Shin JW Verbeke CS Allison KH Extracellular matrix stiffness and composition jointly regulate the induction of malignant phenotypes in mammary epithelium. *Nat Mater.* (2014) 13:970–8. 10.1038/nmat4009 24930031

[29] Marshall SM. 60 years of metformin use: a glance at the past and a look to the future. *Diabetologia.* (2017) 60:1561–5. 10.1007/s00125-017-4343-y 28776085

[30] Zhou J Massey S Story D Metformin LL. An old drug with new applications. *Int J Mol Sci.* (2018) 19:2863. 10.3390/ijms19102863 30241400 PMC6213209

[31] Babcook MA Sramkoski RM Fujioka H Daneshgari F Almasan A Shukla S Combination simvastatin and metformin induces G1-phase cell cycle arrest and Ripk1- and Ripk3-dependent necrosis in C4-2B osseous metastatic castration-resistant prostate cancer cells. *Cell Death Dis.* (2014) 5:e1536. 10.1038/cddis.2014.500 25412314 PMC4260755

[32] Feng Y Ke C Tang Q Dong H Zheng X Lin W Metformin promotes autophagy and apoptosis in esophageal squamous cell carcinoma by downregulating Stat3 signaling. *Cell Death Dis.* (2014) 5:e1088. 10.1038/cddis.2014.59 24577086 PMC3944271

[33] Zhang Y Zheng L Ding Y Li Q Wang R Liu T MiR-20a induces cell radioresistance by activating the PTEN/PI3K/Akt signaling pathway in hepatocellular carcinoma. *Int J Radiat Oncol Biolo Phys.* (2015) 92:1132–40. 10.1016/j.ijrobp.2015.04.007 26031366

[34] Xin X Wu M Meng Q Wang C Lu Y Yang Y Long noncoding RNA HULC accelerates liver cancer by inhibiting PTEN via autophagy cooperation to miR15a. *Mol Cancer.* (2018) 17:94. 10.1186/s12943-018-0843-8 29895332 PMC5998602

[35] Huang CF Teng YH Lu FJ Hsu WH Lin CL Hung CC beta-mangostin suppresses human hepatocellular carcinoma cell invasion through inhibition of MMP-2 and MMP-9 expression and activating the ERK and JNK pathways. *Environ Toxicol.* (2017) 32:2360–70. 10.1002/tox.22449 28722351

[36] Levental KR Yu H Kass L Lakins JN Egeblad M Erler JT Matrix crosslinking forces tumor progression by enhancing integrin signaling. *Cell.* (2009) 139:891–906. 10.1016/j.cell.2009.10.027 19931152 PMC2788004

[37] Valastyan S Weinberg RA. Roles for microRNAs in the regulation of cell adhesion molecules. *J Cell Sci.* (2011) 124(Pt 7):999–1006. 10.1242/jcs.081513 21402873 PMC3056602

[38] Moro A Driscoll TP Boraas LC Armero W Kasper DM Baeyens N MicroRNA-dependent regulation of biomechanical genes establishes tissue stiffness homeostasis. *Nat Cell Biol.* (2019) 21:348–58. 10.1038/s41556-019-0272-y 30742093 PMC6528464

[39] Mouw JK Yui Y Damiano L Bainer RO Lakins JN Acerbi I Tissue mechanics modulate microRNA-dependent PTEN expression to regulate malignant progression. *Nat Med.* (2014) 20:360–7. 10.1038/nm.3497 24633304 PMC3981899

[40] Gu J Wang D Zhang J Zhu Y Li Y Chen H GFRalpha2 prompts cell growth and chemoresistance through down-regulating tumor suppressor gene PTEN via Mir-17-5p in pancreatic cancer. *Cancer Lett.* (2016) 380:434–41. 10.1016/j.canlet.2016.06.016 27400681

