# Supplementary material for: Matrix Stiffness-Upregulated MicroRNA-17-5p Attenuates the Intervention Effects of Metformin on HCC Invasion and Metastasis by Targeting the PTEN/PI3K/Akt Pathway
Source: Front Oncol. 2020 Aug 19;10:1563. doi: 10.3389/fonc.2020.01563 (PMC7466473; doi:10.3389/fonc.2020.01563)
Supplement: FIGURE S1 — Schematic diagram of metformin intervention on HCC cells grown on different-stiffness substrates. [file Presentation_1.pdf]

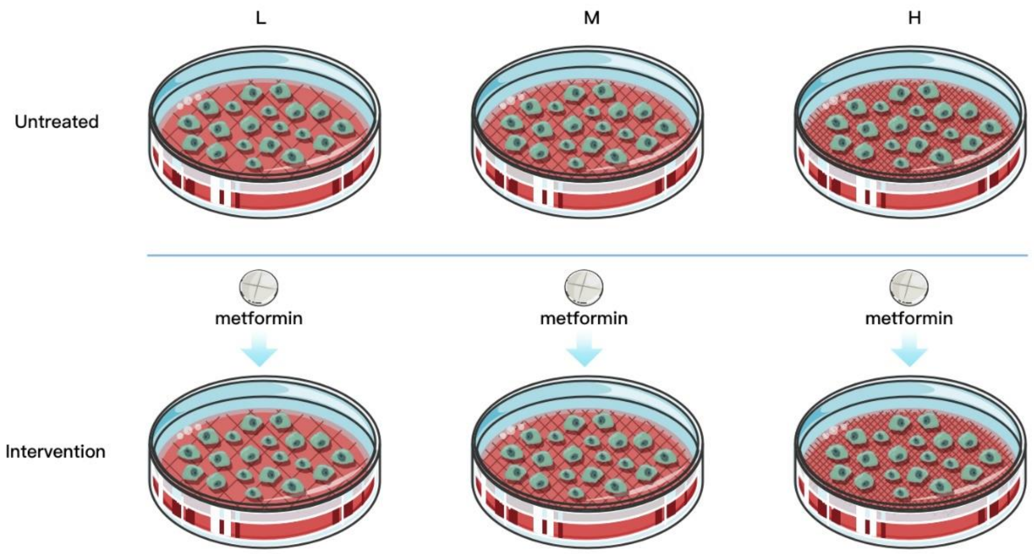

Fig.S1 Schematic diagram of metformin intervention on HCC cells grown on different stiffness substrates.

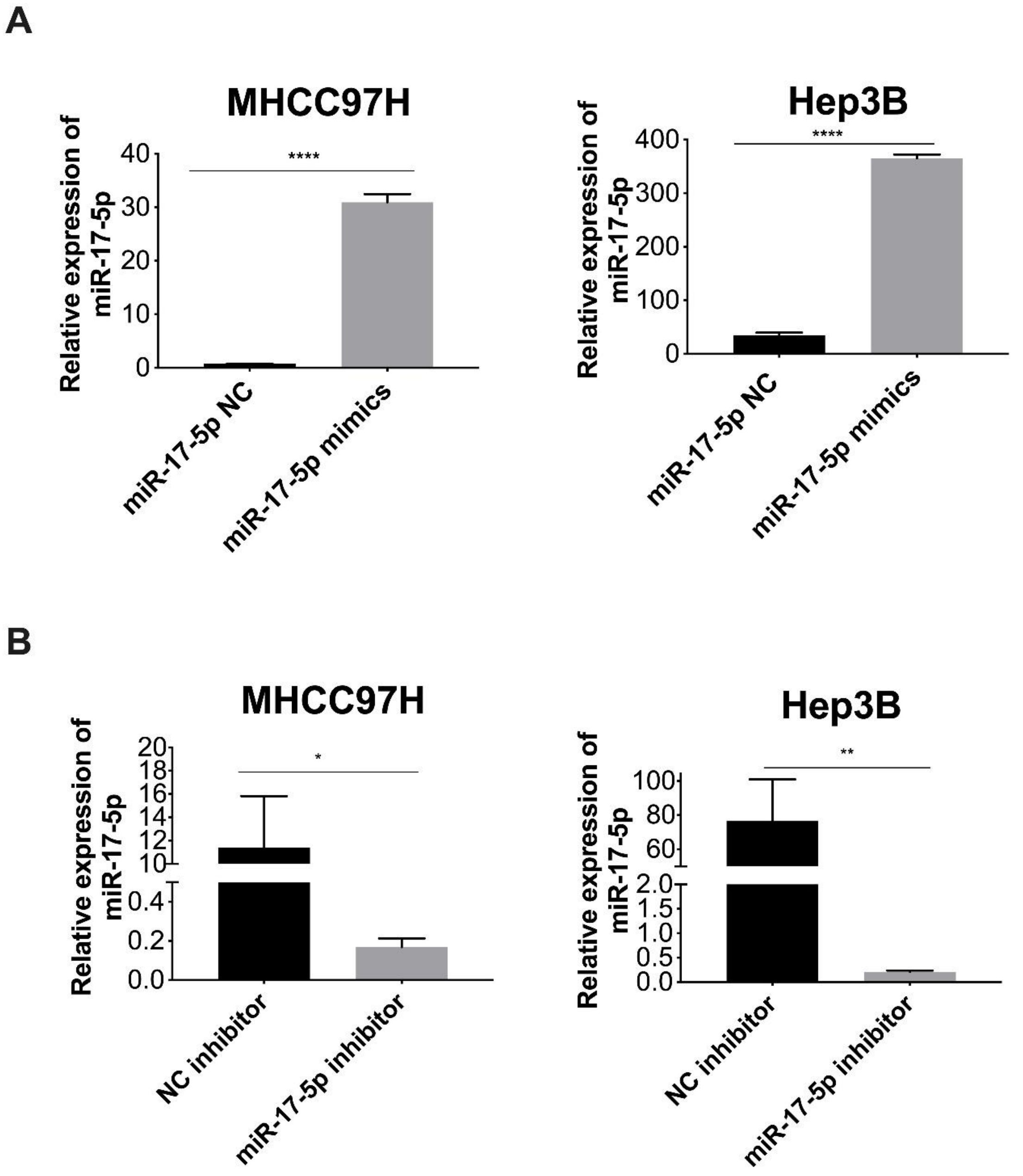

Fig.S2 Assessment of miRNA-17-5p transfection efficiency. (A) MiRNA-17-5p mimic (50nmol/l) transient transfection in MHCC97H and Hep3B cells. (B) MiRNA-17-5p inhibitor (50nmol/l) transient transfection in MHCC97H and Hep3B cells. Error bar represents a standard error of the means (SEM), *p<0.05, **p<0.01, ***p<0.001 and ****p<0.0001.

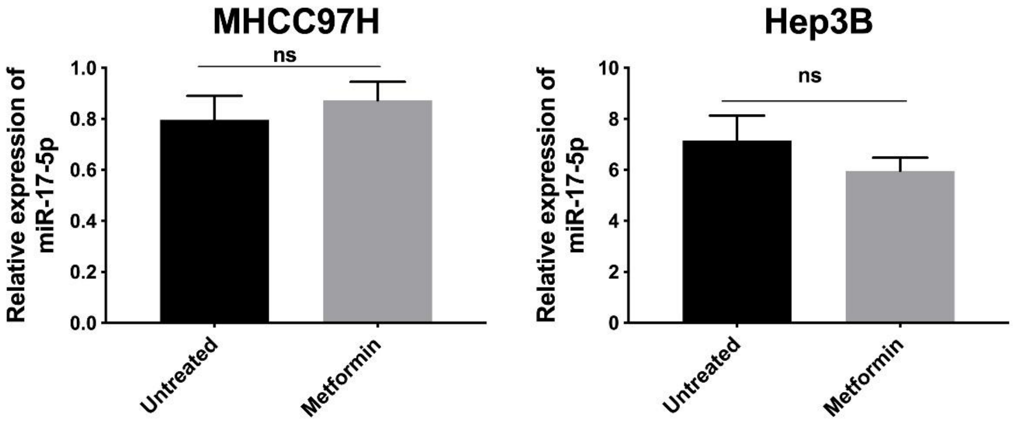

Fig.S3 Metformin has little effect on miR-17-5p expression level in HCC cells (27mM for MHCC97H cells and 22mM for Hep3B cells for 24h). Error bar represents a standard error of the means (SEM), *p<0.05, **p<0.01, ***p<0.001 and ****p<0.0001.

Table S1 Quantitative density area value of immunohistochemistry in HCC tissue microarray

	L	M	H
PTEN	30.54	27.34	23.76
	30.81	30.16	24.99
	33.14	29.56	25.84
	32	32.9	24.61
	31.63	27.7	21.82
	33.26	26.97	26.53
	34.72	27.5	20.83
	38.47	27.42	23.34
	36.8	25.19	19.32
	44.33	26.98	23.36
	35.9	32.03	26.56
	45.37	25.7	19.08
MMP2	23.74	41.68	29.4
	20.47	30.71	29.25
	21.65	32.76	32.54
	22.62	28.84	32.81
	22.65	22.95	29.92
	23.85	26.02	33.08
	23.37	29.72	28.32
	20.08	28.55	31.38
	20.49	30.05	30.77
	21.57	29.31	31.25
	23.38	28.31	29.79
	20.01	29.09	32.07
MMP9	23.17	32.94	44.54
	21.37	36.08	38.81
	12.38	31.72	48.89
	19.53	38.66	39.62
	25.48	35.84	39.89
	26.48	33.18	48.36
	32.85	50.09	56.74
	35.67	39.87	47.95
	30.58	42.6	62.94
	29.6	39.33	56.16
	32.98	41.7	56.08
	34.29	38.2	48.21

Table S1 Quantitative density area value of immunohistochemistry in HCC tissue microarray.
